# Tracking Lead: Potentiometric Tools and Technologies for a Toxic Element

**DOI:** 10.3390/molecules30173492

**Published:** 2025-08-25

**Authors:** Martyna Drużyńska, Nikola Lenar, Beata Paczosa-Bator

**Affiliations:** Faculty of Materials Science and Ceramics, AGH University of Krakow, Mickiewicza 30, 30059 Krakow, Poland; druzynska@agh.edu.pl

**Keywords:** lead determination, ion-selective electrodes, potentiometry, Pb^2+^ detection, environmental monitoring, electrochemical sensors

## Abstract

Lead contamination remains a critical global concern due to its persistent toxicity, bioaccumulative nature, and widespread occurrence in water, food, and industrial environments. The accurate, cost-effective, and rapid detection of lead ions (Pb^2+^) is essential for protecting public health and ensuring environmental safety. Among the available techniques, potentiometric sensors, particularly ion-selective electrodes (ISEs), have emerged as practical tools owing to their simplicity, portability, low power requirements, and high selectivity. This review summarizes recent progress in lead-selective potentiometry, with an emphasis on electrode architectures and material innovations that enhance analytical performance. Reported sensors achieve detection limits as low as 10^−10^ M, broad linear ranges typically spanning 10^−10^–10^−2^ M, and near-Nernstian sensitivities of ~28–31 mV per decade. Many designs also demonstrate reproducible responses in complex matrices. Comparative analysis highlights advances in traditional liquid-contact electrodes and modern solid-contact designs modified with nanomaterials, ionic liquids, and conducting polymers. Current challenges—including long-term stability, calibration frequency, and selectivity against competing metal ions—are discussed, and future directions for more sensitive, selective, and user-friendly Pb^2+^ sensors are outlined.

## 1. Introduction

**Figure 1:**
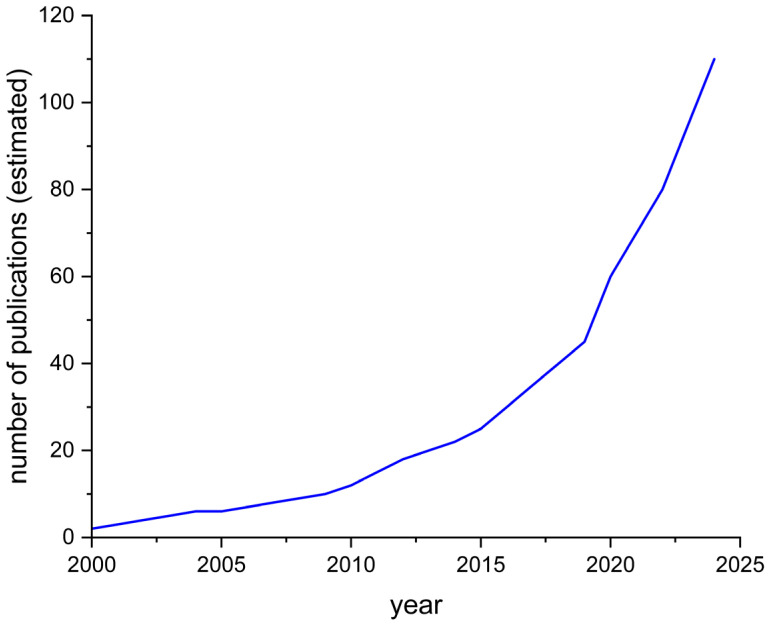
Estimated trend in the number of publications indexed in PubMed (2000–2024) related to potentiometric ion-selective electrodes for lead(II) determination. The data are illustrative and based on a bibliometric trend analysis rather than exact counts.

Lead (Pb) is a toxic heavy metal that poses a significant threat to human health and the environment. Despite increasing global awareness and regulatory efforts to reduce lead exposure, it remains widespread due to its extensive use in industrial processes, batteries, pigments, ceramics, and plumbing materials. Lead pollution in the environment arises both from natural processes such as volcanic eruptions and the weathering of rocks, as well as from anthropogenic sources including mining, battery production, fertilizers, ceramics, and wastewater discharge. Once released, lead persists in air, soil, and water, where it accumulates and enters the food chain. For humans, exposure even at trace levels is associated with severe health risks, including neurodevelopmental disorders in children, renal impairment, hypertension, and cardiovascular diseases. The bioaccumulative and persistent nature of lead makes it a long-term threat for both environmental and public health. Even at low concentrations, lead exposure can cause severe health issues, including neurotoxicity, renal impairment, anemia, and developmental delays in children. As such, the reliable detection and quantification of lead in various matrices—such as water, soil, food, and biological samples—are of crucial importance [1,2,3,4,5,6]. Conventional analytical methods of lead determination, such as atomic absorption spectrometry (AAS), inductively coupled plasma mass spectrometry (ICP-MS), and anodic stripping voltammetry (ASV), offer high sensitivity and accuracy. However, they often require complex instrumentation, skilled personnel, and extensive sample preparation, which can limit their use in field settings or routine monitoring applications [7]. Potentiometry, particularly through the use of ion-selective electrodes (ISEs), presents a viable alternative. This technique allows for the direct, on-site measurement of ionic activity with minimal sample processing. Therefore, compared with traditional spectrometric and electroanalytical methods, ISE-based potentiometric sensors stand out not only for their operational simplicity but also for the recent improvements in their detection limits, stability, and applicability in real environmental and food matrices. Potentiometric sensors designed specifically for lead ions (Pb^2+^) have shown promising performance in terms of selectivity, detection limits, and operational simplicity. These sensors operate by converting the activity of the target ion into an electrical potential, which can be measured against a reference electrode under near-equilibrium conditions [8,9,10,11,12]. In recent years, the field of potentiometric lead sensing has undergone substantial development. As shown in Figure 1, the number of publications on lead(II) ion-selective electrodes has steadily increased since 2000, with a marked acceleration after 2015 and a sharp rise in recent years (2020–2024).

While conventional ISEs with an internal solution demonstrated reliable performance, modern designs—particularly solid-contact electrodes (SCEs)—have achieved remarkable progress in sensitivity, stability, and practical applicability. By incorporating nanomaterials, conducting polymers, and ionic liquids into electrode membranes and transducer layers, researchers have reported detection limits down to 10^−10^ M, near-Nernstian slopes of 28–31 mV per decade, and broad linear ranges extending from 10^−10^ to 10^−2^ M. Several of these sensors have been successfully applied to complex matrices including wastewater, seawater, and food products, demonstrating their potential as real-world monitoring tools. These innovations are driving the evolution of potentiometric sensors from laboratory tools to robust, field-deployable devices. This review focuses on the potentiometric determination of lead using ion-selective electrodes. It covers the fundamental principles of potentiometric sensing, reviews the toxicological significance of lead, and explores various electrode designs and sensing strategies. Special attention is given to the challenges currently faced in this area and the prospects for developing more sensitive, selective, and user-friendly potentiometric sensors for lead analysis.

## 2. Toxicity of Lead

### 2.1. Sources of Lead Pollution

**Figure 2:**
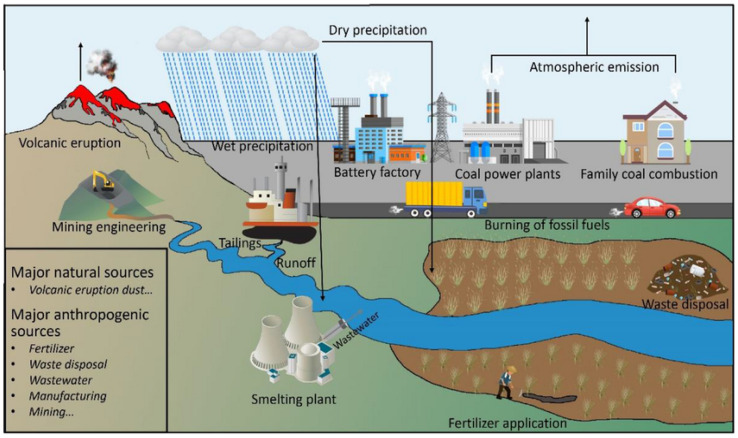
Sources of lead pollution in the environment. Reprinted from [19] with the permission of Springer Nature BV.

Lead (Pb) is an element classified as heavy metal due to its high toxicity to organisms even at small concentrations [13,14,15,16]. Environmental lead pollution affects air, soil, and water quality [17,18]. The presence of lead in the environment has natural origins such as volcano eruptions or geochemical weathering [17,19], but most of the contamination is strictly connected with human industrial activity. The sources of pollution include urbanization [20], mining, fertilization [21], glass industry [22], and wastewater discharge [23,24]. This highly toxic metal can be found in paint, gasoline, batteries, cutlery, jewelry, or even toys [15,25,26]. A schematic representation of lead emission sources into the environment is shown in Figure 2.

### 2.2. Effects on Environment

Lead is a persistent element that builds up in water ecosystems and soil. These properties make it a serious threat to the environment [27]. When lead accumulates in soil used for growing plants, it can disturb plant functions, as it mainly stays in the roots [28,29]. Plants in contaminated areas may show reduced seedling height, lower photosynthesis efficiency, and problems with metabolism due to changes in hormone levels [30,31,32,33,34]. In water, lead harms both aquatic life and human health. It can also cause economic losses and slow down development by polluting water, making it harder and more expensive to use.

### 2.3. Effects on Human

Lead, as a heavy metal, is especially harmful for humans. This element is classified as a carcinogen. It also exhibits high neurological toxicity, especially for children. Lead accumulates in bones, interfering with nerve and muscle function. Research has shown [26,35,36,37,38] that children who were exposed to lead show signs of brain dysfunction and they can suffer from behavioral disorders. Beyond its general toxicity, Pb^2+^ exhibits pronounced neurotoxic effects. One of the main mechanisms involves interference with calcium homeostasis: Pb^2+^ can enter voltage-gated Ca^2+^ channels and substitute for calcium ions, thereby disrupting neurotransmitter release and synaptic communication. In addition, Pb^2+^ exposure increases oxidative stress by generating reactive oxygen species (ROS) and weakening antioxidant defenses, which leads to lipid peroxidation, mitochondrial dysfunction, and ultimately neuronal injury. Furthermore, chronic exposure has been linked to neuroinflammatory responses and altered gene expression in the central nervous system. These mechanisms collectively impair neurodevelopment, resulting in cognitive deficits, learning difficulties, and behavioral disorders such as hyperactivity and aggression. Recent studies confirm that Pb^2+^ is not only a developmental neurotoxicant but also contributes to long-term neurological and metabolic alterations [5].

## 3. Background of Potentiometric Determination of Metal Ions

### 3.1. Potentiometry Basics

**Figure 3:**
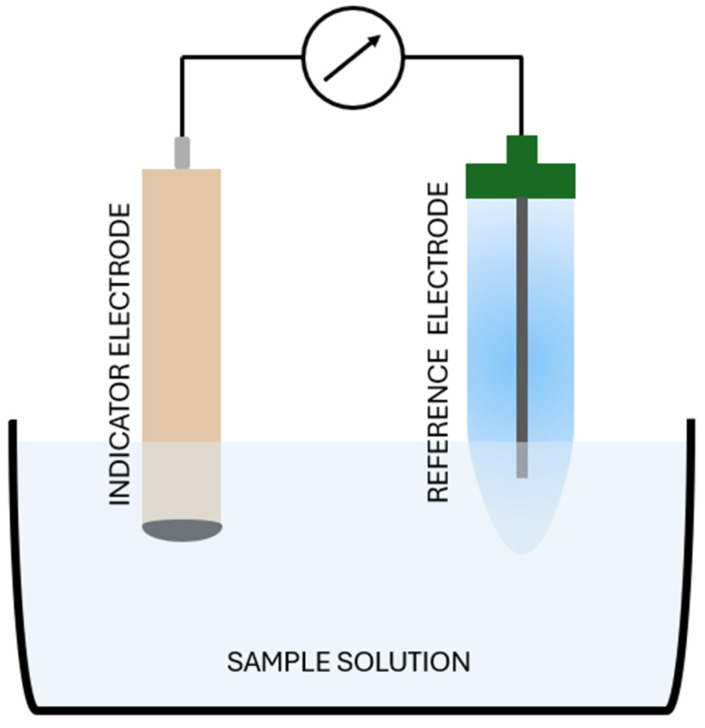
Schematic representation of a potentiometric cell.

Potentiometry is one of the oldest instrumental methods of chemical analysis. In this method, the electromotive force (EMF) of a cell is measured under zero-current conditions. A typical potentiometric cell containing indicator and reference electrodes is shown in Figure 3. The primary factor influencing the EMF value is the electrode potential, which depends on the nature of the electrode processes and the activity of the ions present in the solution in which the electrodes are immersed. The determination of a given component is based on the relationship between the electric potential of the indicator electrode and the activity of the ions present in the sample, relative to the reference electrode, whose potential is known and remains unchanged. This relationship for reference electrodes can be described by the Nernst Equation (1) [39,40,41]:

(1) $$E=E^{0}-\frac{RT}{zF}\mathrm{lna}$$

where E stands for the measured potential of the ISE cell,$\;E^{0}$ stands for the standard electrode potential, R stands for the ideal gas constant, T stands for temperature, z  stands for the charge number of the potential-determining ion, F  stands for Faraday’s constant, and a  stands for the activity of the potential-determining ion.

### 3.2. Ion-Selective Electrodes

Ion-selective electrodes (ISEs) are a robust tool for potentiometric ion analysis. The development of ion-selective electrodes began in 1906, when M. Cremer observed that certain types of glass are sensitive to changes in hydrogen ion concentration [42]. Ion-selective electrodes are defined as electrochemical sensors with a potential linearly dependent on the logarithm of the activity of the measured ion in the solution. A fundamental principle of ion-selective electrode operation is the conversion of the electrochemical interaction between the target ion and the membrane into an electrical signal. For ISEs, the Nikolsky–Eisenman equation is commonly applied to account for the electrode’s response not only to the primary ion but also to interfering ions present in the solution [43].

(2) $$E=E^{0}\pm 2.303\frac{RT}{zF}log(a_{I}+\sum_{I\neq J}K_{IJ}a_{J}^{\frac{z_{I}}{z_{J}}}+a^{0})$$

where E stands for the measured potential of the ISE cell,$\;E^{0}$ stands for the standard electrode potential, R stands for the ideal gas constant, T stands for temperature, $z_{I}\;$ stands for the charge of the primary ion, $z_{J}$ stands for the charge of the interfering ion, $a_{I}$ stands for the activity of the primary ion, $a_{J}\;$ stands for the activity of the interfering ion, F  stands for Faraday constant, and $K_{IJ}$ stands for the selectivity coefficient.

### 3.3. Potentiometric Titration

Potentiometric titration has also been employed as a complementary approach to Pb^2+^ determination. In such methods, Pb^2+^-selective electrodes serve as indicator electrodes to monitor the potential change during titration with suitable complexing agents, most commonly EDTA. This strategy provides distinct inflection points that allow reliable end-point detection even in the presence of background electrolytes, and it can achieve detection limits in the micromolar to sub-micromolar range. In those methods, Pb^2+^-selective electrodes serve as indicator electrodes to track potential changes during titration with EDTA. For example, a poly(m-phenylenediamine)-based ISE demonstrated high selectivity towards Pb^2+^ and was used successfully as an indicator electrode in titrations of Pb^2+^ with EDTA, even in challenging real-world samples such as urine and river water [44]. Similarly, a PVC-based membrane electrode incorporating a thiacrown ionophore proved effective as an indicator electrode for Pb^2+^ titration in water samples [45]. Compared with direct potentiometric measurements, titration-based approaches offer improved accuracy in samples with high ionic strength or complex matrices, although they are less suitable for real-time or on-site applications. Recent studies have demonstrated that potentiometric titration with ion-selective electrodes remains a valuable tool for validating direct Pb^2+^ measurements and for assessing the robustness of newly developed electrode designs in practical sample conditions.

In addition to aqueous systems, potentiometric titration of Pb^2+^ has also been explored in non-aqueous or mixed-solvent environments. Organic solvents can influence electrode response by altering ion activity and dielectric properties, which is particularly relevant when natural samples contain high levels of organic matter. Non-aqueous potentiometry is related to challenges compared to water-based systems: differences in ion activity, dielectric constant, and reference electrode performance can lead to deviations in calibration slope and endpoint detection, as well as altered electrode stability [46]. Despite these difficulties, several Pb^2+^-selective electrodes have demonstrated applicability in media containing organic solvents. For example, Schiff base-based Pb^2+^ ISEs retained stable and reproducible responses in matrices containing up to 15% (*v*/*v*) non-aqueous content, showing their potential in samples with organic interference [47]. Furthermore, manufacturers such as Metrohm report that crystal membrane Pb^2+^ electrodes can be applied short-term in organic solvents such as acetone, methanol, or benzene, although with caution regarding long-term stability [48]. These findings underline that while aqueous systems remain the most common application domain, extending potentiometric Pb^2+^ analysis into non-aqueous environments is feasible and valuable, particularly for samples rich in organic matter such as industrial effluents or organic-contaminated wastewaters.

## 4. Ion-Selective Electrodes for Lead Detection

Lead-selective ion-selective electrodes (ISEs) have been known and commercially available for years. They are used in the direct analysis of lead content in samples, as well as in the titration of sulfates using solutions containing lead(II) ions. ISEs reliably sense Pb^2+^ ions. However, various hydroxyls may be present in aquatic lead solution with different pH levels. Awareness of the presence of lead ions in forms different to Pb^2+^ is important in studies of analytical parameters and determining working pH range of an electrode [49,50].

P. Kivalo et al. carried out an evaluation of lead-selective electrodes that were commercially available in the 1970s. The valuation included tests aimed at determining analytical parameters of the electrodes, such as the limit of detection sensitivity and selectivity [51].

The first type of electrode was Orion Research Inc., lead electrode 94-82. The experiments have shown that, over the course of calibration, the electrode exhibits a near-Nernstian response, around 29 mV per decade. The detection limit obtained for the Orion electrodes ranges from 10.5 to 11.5 (−log_cPb_^2+^). The sensors exhibit stable potentiometric response until pH 6 is reached for a 10^−2^ M concentration of lead(II) ions. Up to pH 6, the change in pH has no effect on the potential of Orion Research Inc. (Cambridge, MA, USA) lead electrodes [51].

Another type of electrode examined in the study was the Radiometer A/S, Ruzicka Selectrode Kit 3012 with lead Selectrode powder S 42215. The experimental results demonstrated that, during calibration, the electrode displayed a sub-Nernstian slope of approximately 25 mV per decade. The detection limit for the Ruzicka Selectrodes was found to be between 10.5 and 11.5 (–log_cPb_^2+^). Variations in pH did not affect the electrode potential. A stable potentiometric response was observed up to pH 6 for a lead(II) ion concentration of 10^−2^ M [51].

Currently, Metrohm is one of the companies offering a commercially available ion-selective electrode specifically designed for detecting lead(II) ions. According to the manufacturer, lead-selective electrode 6.0502.170 can be successfully used in direct ion measurements of Pb^2+^ and titrations. The electrode allows for the determination of lead ion concentrations in the range of 10^−6^ to 10^−1^ M. Change in pH has no effect on the potential of this kind of lead electrode in the range of 4–7. The electrode shaft is made of epoxy resin, providing high mechanical durability. The electrode can be used at temperatures up to 80 °C [52]. 

Orion is another supplier of ion-selective electrodes constructed for the detection of lead(II) ions. The model 9682BNWP, according to the manufacturer, is effective for direct Pb^2+^ ion analyses. It operates reliably within a concentration range of 10^−6^ to 10^−1^ M. The electrode is suitable for use at temperatures up to 80 °C. The electrode should be stored in a calibration solution containing 0.1 M Pb(ClO_4_)_2_ and 0.1 M Na_2_SO_4_ [53]. The lead-selective electrode produced by Orion is suitable for evaluating the performance of newly synthesized membranes in water purification processes [54].

**Figure 4:**
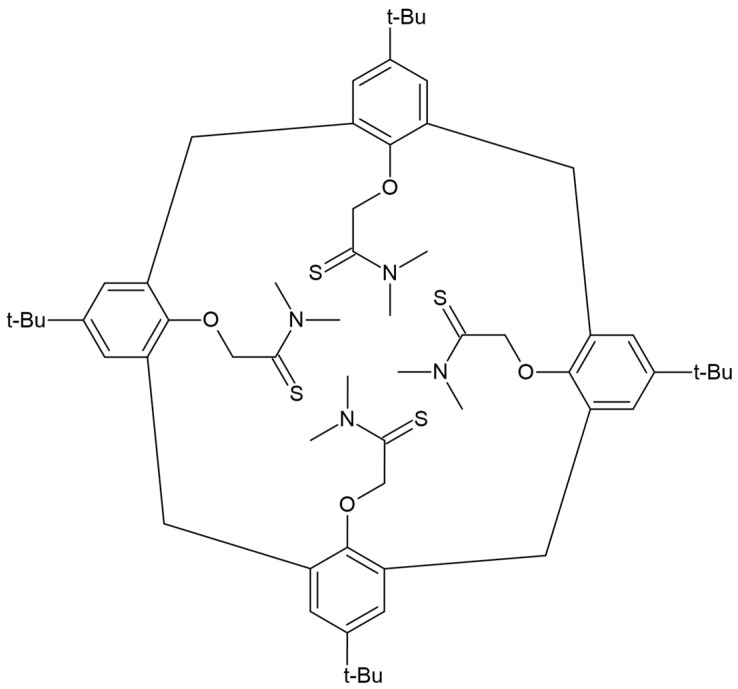
Lead ionophore IV molecule.

Conventional ion-selective electrodes with an internal solution turned out to be a reliable tool for the detection of Pb^2+^ ions; however, among the various electrode designs, solid-contact electrodes (SC) have gained the most popularity due to their simplicity and ease of miniaturization. To ensure proper performance, this type of ion-selective electrode, as well as electrodes of a classical design with an inner solution, must exhibit high selectivity, a long lifetime, and a stable potential response over time [55,56,57,58]. Most ISEs rely on the presence of an ionophore—an active compound capable of selectively complexing target ions. Several compounds can serve as effective ionophores for lead ions. The most popular and commercially available lead ionophore is tert-Butylcalix[4]arene-tetrakis(N,N-dimethylthioacetamide), known as lead ionophore IV. It functions as an ionophore due to its structure—sulfur atoms with a double bond show high affinity with Pb^2+^ cations. However, this ionophore shows poor selectivity over silver ions, which typically bind even more strongly. The lead ionophore IV molecule is shown in Figure 4 [12,49,59].

**Figure 5:**
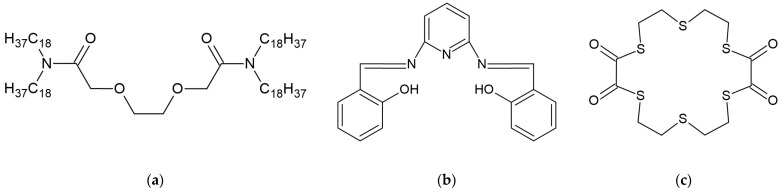
Examples of compounds used as lead ionophores: (**a**) derivative of oxy-diamide; (**b**) derivative of Schiff base; (**c**) derivative of crown ether.

Other compounds have also been employed as lead ionophores. Among them, oxy-diamides [49,60], Schiff bases [61,62], and crown ethers [62] can be distinguished. A detailed description of the molecular properties and derivatives of the compounds used as lead-selective ionophores was provided by M. Guziński et al. [12]. Examples of compounds used as lead ionophores are shown in Figure 5.

## 5. Lead-Selective Electrode Design Solutions

### 5.1. Electrode with Internal Solution

A conventional electrode with internal solution is a common type of ion-selective electrode used in potentiometry. It consists of an ion-selective membrane, an internal reference electrode (usually silver/silver chloride), and an internal filling solution that maintains stable conditions inside the electrode. The internal solution allows for ion exchange across the membrane and reliable potential measurements. While these electrodes are well-established and offer high accuracy, they can be bulky and are more prone to issues like leakage or evaporation of the internal solution compared to solid-contact designs [56,63].

**Figure 6:**
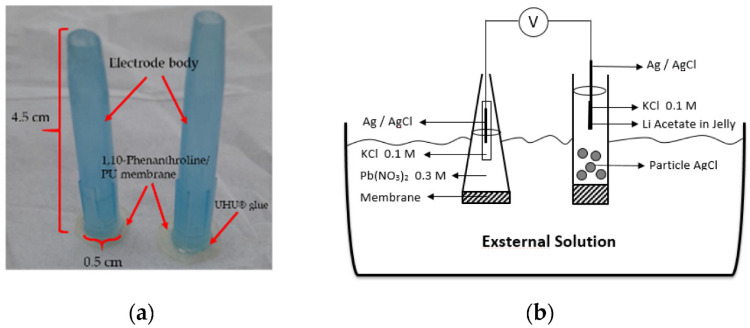
Pb^2+^ ion-selective electrode based on polyurethane (PU) membrane. (**a**) Representation of an electrode, (**b**) Schematic representation of the potentiometric cell [64].

K. Nisah et al. presented a type of ion-selective electrode with an internal solution. The electrode design is based on a castor oil–based polyurethane (PU) membrane, using 1,10-phenanthroline as the active component. To prepare the membrane matrix, toluene diisocyanate (TDI) was mixed with castor oil and stirred briefly. Next, 1,10-phenanthroline was added in varying amounts, and the mixture was stirred until uniform, then gently heated. Acetone was gradually added during sonication to further process the mixture. The solution was spread onto a glass plate and dried in an oven to form the membrane. The prepared membrane was cut into a circular form and attached to the surface of the electrode body. After that, an internal solution containing 0.1 M KCl and 0.3 M Pb(NO_3_)_2_ was added. Before measurements, the ion-selective electrode was then conditioned in a 0.1 M Pb(NO_3_)_2_ solution for 24 h. The sensitivity of the ISE obtained is 27.25 mV per decade with a linear range toward Pb^2+^ ions of 10^−10^–10^−5^ M. The electrode exhibits a limit of detection of 10^−10^ M. The working pH range for this kind of sensor is 7–8. The sensor can be used for lead detection in wastewater. However, a decline in its mechanical properties was observed after the measurements [64]. The electrode project is presented in Figure 6.

### 5.2. Carbon Paste Electrode

A carbon paste electrode (CPE) is a type of working electrode commonly used in potentiometry and other electrochemical methods. It is made by mixing paste components (e.g., graphene or carbon black) with a binder, usually a liquid like mineral oil or paraffin, to form a paste. This paste is packed into an electrode body and can be easily renewed or modified. CPEs are popular due to their low cost, ease of preparation, chemical stability, and ability to incorporate selective substances, making them useful for detecting specific ions or molecules in solution [65,66].

**Figure 7:**
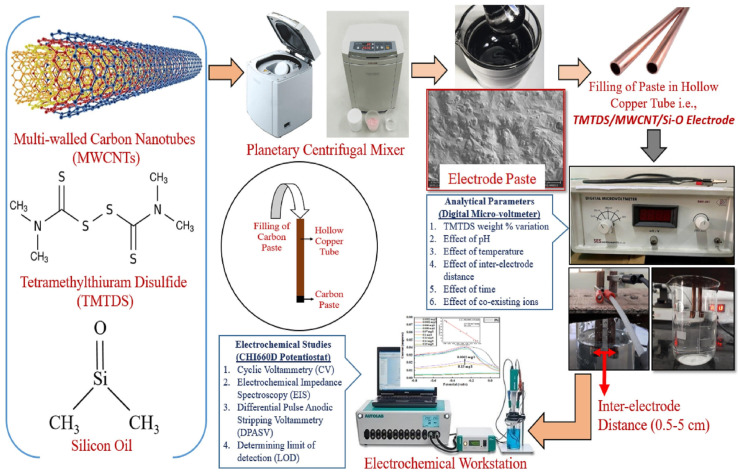
Visual depiction of the procedure of electrode preparation and its use. Reprinted from [67] with the permission of Elsevier.

D. Mishra et al. presented a composite carbon paste electrode for potentiometric lead detection. The electrode project relies on a composite paste containing an ionophore, multi-walled carbon nanotubes bonded by silicon oil. Tetramethyl thiuram disulfide (TMTDS) was used as an ionophore. A copper tube was chosen as an electrode body because of its high thermal and electrical conductivity. Electrode fabrication is straightforward and consists of filling the copper tube with the prepared electrode paste. Prepared sensors were conditioned for 24 h in an aqueous solution with a lead concentration of 0.1 μg/L. Research on the sensor was conducted at various concentrations of TMTDS in order to select the most optimal paste composition. The highest sensitivity was achieved at a 24 wt% ionophore concentration. Experiments have shown that electrodes are sensitive to lower lead concentrations. The sensor exhibits a limit of detection of 31 μg/L. Change in pH has no significant effect on the potential up to pH 5. In solutions of a higher pH, potential increases drastically. The presented sensor is suitable for detection of lead ions in the range of 0.2–150 μg/L. The scheme of the fabrication procedure and the conducted experiments is shown in Figure 7 [67].

Another electrode design based on carbon paste was presented by K. M. Hussein et al. The Teflon holder was used as an electrode body. Two kinds of electrodes were prepared, using different plasticizers. The electrode pastes contain Novel Schiff base ligand ionophore (HPIMN), graphite powder, and TCP (electrode I) or o-NPOE (electrode II) as plasticizers. The obtained electrodes exhibit a near-Nernstian response (around 29 mV er decade) over concentration ranges of 1.0 × 10^−5^–1.0 × 10^−2^ (Electrode I) and 1.0 × 10^−6^–1.0 × 10^−2^ mol/L (Electrode II). Electrode I exhibits a limit of detection of 3.33 × 10^−5^ and 3.33 × 10^−6^ was obtained for electrode II. Change in pH has no effect on the potential of the electrodes in the range of 3–8. The recommended temperature for working with the electrodes should not exceed 50 °C, as higher temperatures may disrupt the course of electrode reactions. The proposed electrodes can be successfully used to measure lead(II) ions concentration in real life samples, such as wastewater, hazelnut, carrot, mushroom, and tuna [68].

### 5.3. Solid-Contact Electrodes

A solid-contact electrode (SCE) is another type of ion-selective electrode that eliminates the need for an internal liquid solution. Instead, it uses a solid material to form the contact between the ion-selective membrane and the electronic conductor. Solid-contact electrodes offer good mechanical stability and are less prone to leakage or evaporation, making them ideal for long-term and field measurements [69,70,71].

A. Jasinski et al. presented a different approach to preparing a lead-selective electrode by introducing a solid-contact electrode with a polymeric ion-selective membrane. The designed sensor is intended for the determination of lead in the presence of high concentrations of interfering metal ions. The membrane cocktail was prepared using 1.1 wt% of ionophore (25,26,27,28-tetrakis(piperidinylthiocarbonylmethylene)-p-tert-butylcalix[4]arene, 32.5 wt% PVC, 65 wt% o-NPOE or DOS as plasticizer and 0.25 wt% of KTpClPB as lipophilic salt. The membrane components were dissolved in tetrahydrofuran (THF). Poly(3,4-ethylenedioxythiophene) (PEDOT) doped with poly(styrene sulfonate) (PSS) were used as a solid contact material and placed directly onto a previously polished and cleaned GC disk electrode. The membranes were cast onto the surface of the previously prepared GC/PEDOT(PSS) electrodes using a drop-casting technique, with a volume of 40 µL. Before measurements, prepared sensors were conditioned in 10^−4^ M Pb(NO_3_)_2_ for 12 h. The findings indicate that solid-contact lead-selective electrodes can serve as an effective tool for detecting lead(II) ions in environmental samples containing high levels of interfering ions like Na^+^, Zn^2+^, Cu^2+^, Cd^2+^, Mg^2+^, or Ca^2+^ [11].

A different type of solid-contact electrode for lead detection was presented by C. Wardak. In this research, ionic liquids were introduced as additional components for ion-selective PVC-based membranes. 1-ethyl-3-methylimidazolium chloride (EMImCl), 1-butyl-3-methylimidazolium chloride (BMImCl), 1-hexyl-3-methylimidazolium chloride (HMImCl), 1-decyl-3-methylimidazolium chloride (DMImCl), and 1-benzyl-3- methylimidazolium chloride (NMImCl) were introduced as additive ionic liquids. The lead-selective membrane was composed of the following components: 2-Nitrophenyl octyl ether (o-NPOE), bis(1-butylpentyl) adipate (BBPA), tributyl phosphate (TBP), poly(vinyl chloride) (PVC), tert-butylcalix[4]arene-tetracis(N,N-dimethylthioacetamide) (lead ionophore IV). The electrode membrane comprised two layers assembled within a Teflon holder. The inner layer consists of plasticized PVC doped with an ionic liquid in which the Ag/AgCl electrode was placed. The outer layer, containing an ionophore along with the same components as the inner layer, was positioned in direct contact with the test solution. The prepared electrode was conditioned before the measurements in an appropriate solution of Pb(NO_3_)_2_. The developed electrode exhibits a clear Nernstian response toward Pb^2+^ ions across a broad concentration range from 1 × 10^−8^ to 1 × 10^−1^ M, with a slope of 29.8 mV per decade. The detection limit was determined to be 4.3 × 10^−9^ M, indicating high sensitivity. Its performance remains unaffected within the pH range of 3.5 to 7.3. The electrode shows excellent selectivity for lead(II) ions, effectively discriminating against common interfering ions. Its practical utility was confirmed through its successful application as an indicator electrode in the potentiometric titration of Pb^2+^ with K_2_CrO_4_, as well as in the direct determination of lead ions in real sample matrices [9]. 

Later, the group of C. Wardak et al. presented a similar, improved electrode design. The ion-selective membrane was modified with a nanocomposite (NC) consisting of the ionic liquid 1-hexyl-3-methylimidazolium hexafluorophosphate (HMImPF_6_) and carbon nanofibers (CNF). The composition of the ion-selective membrane was as follows: lead ionophore (1 wt%), NC (6 wt%), PVC (33 wt%), BBPA (32.5 wt%) o-NPOE (27.5 wt%). The electrode. The electrode exhibits a near-Nernstian response (slope 31.5 mV per decade) over concentration ranges of 1.0 × 10^−5^–1.0 × 10^−2^ with a limit of detection of 6.0 × 10^−9^. The working pH range for this kind of sensor was determined to be 3.1–7.6. Moreover, no redox response was registered in the electrodes. To test the electrode’s response, the lead content was determined in the certified reference material of wastewater [72].

**Figure 8:**
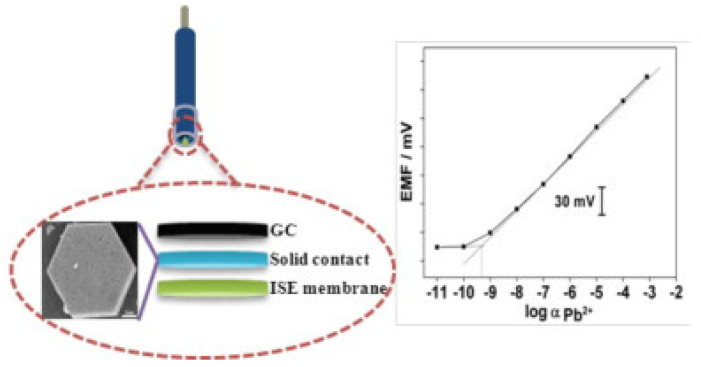
The scheme of the electrode design and the calibration curve. Reprinted from [73] with the permission of Elsevier.

Bimodal pore C_60_ (BP-C_60_) was introduced as solid-contact layer by J. Li et al. In this work, a BP-C_60_ film was electrodeposited on a glassy carbon (GC) electrode. An ion-selective membrane containing lead ionophore IV (1.00 wt%), sodium trakis[3,5-bis(trifluoro-methyl)phenyl]borate (NaTFPB) (0.44 wt%), tetradodecylammonium tetrakis-(4-chlorophenyl)borate (ETH 500) (1.00 wt%), poly(vinyl chloride) (PVC) (32.52 wt%), and 2-nitrophenyl octyl ether (o-NPOE) (65.04 wt%) was drop-cast onto the GC electrode. A near-Nernstian response with a slope of 28.8 per decade was obtained toward lead(II) ions in the range of 1.0 × 10^−9^ to 1.0 × 10^−3^ M. The detection limit was determined to be 5.0 × 10^−10^ M. Prepared sensors were tested and used to determine the lead(II) concentration in tap water. Before measurements, prepared sensors were conditioned in aqueous lead(II) solutions: 10^−3^ M Pb(NO_3_)_2_ for one day and 10^−9^ M Pb(NO_3_)_2_ for two days. The sensor project is presented in Figure 8 [73].

**Figure 9:**
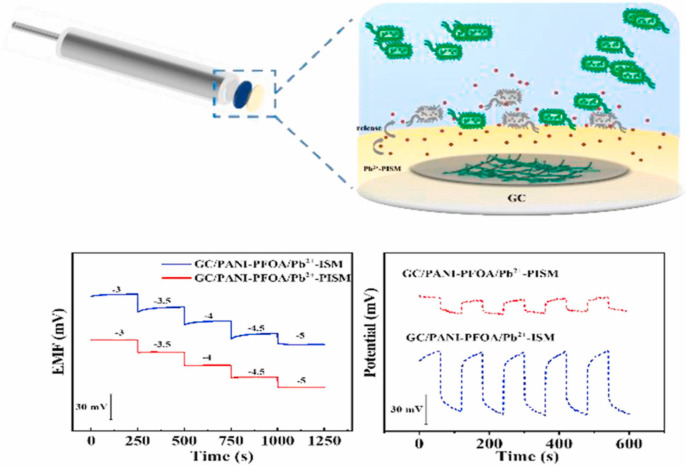
The functioning of the electrode and its analytical performance. Reprinted from [74] with the permission of Elsevier.

Y. Liu et al. presented an improvement in the solid-contact electrode with antibacterial properties [74]. A derivative of capsaicin propyl 2-(acrylamidomethyl)-3,4,5-trihydroxy benzoate (PAMTB) was introduced as an antifouling agent due to its antibacterial activity and environmental friendliness, and was previously reported in studies highlighting its potential in surface protection applications [75,76]. To fabricate a Pb^2+^ selective electrode, the polyaniline-perfluorooctanoic acid (PANI-PFOA) solid-contact layer was deposited onto the GC electrode. The membrane cocktail consisted of lead ionophore IV (1.4 wt%), o-NPOE (63 wt%), PVC (35 wt%), and NaTFPB (0.6 wt%) with the addition of various amounts of PAMTB (1.2 wt%, 1.6 wt%, 2.0 wt%, 2.5 wt%). To fabricate a Pb^2+^-selective electrode, the PANI-PFOA solid-contact layer was deposited onto the GC electrode. The membrane cocktail consisted of lead ionophore IV (1.4 wt%), o-NPOE (63 wt%), PVC (35 wt%), and NaTFPB (0.6 wt%) with the addition of various amounts of PAMTB (1.2 wt%, 1.6 wt%, 2.0 wt%, 2.5 wt%). This sensor is characterized as having excellent antifouling properties while maintaining its analytical performance. The presented ISE exhibit Nernstian response in the Pb(NO_3_)_2_ concentration range of 1.0 × 10^−3^ to 1.0 × 10^−6^ M, with the slope being 28.5 ± 0.8 mV. The obtained limit of detection is 1.9 × 10^−7^ M. The sensor can be successfully used in lead determination in seawater [74]. The design of the electrode and its analytical performance is shown in Figure 9.

Although these three electrode types are already well-established, recent developments have significantly advanced their analytical utility. For instance, internal-solution electrodes now reach detection limits as low as 10^−10^ M, comparable with advanced voltammetric methods. Carbon paste electrodes remain highly attractive due to their low cost and flexibility in incorporating new ionophores, enabling their application in food analysis. Solid-contact electrodes represent the most significant innovation, eliminating internal solutions and introducing nanomaterials, conducting polymers, and ionic liquids to improve signal stability, miniaturization, and field applicability. In contrast to AAS or ICP-MS, which require expensive instrumentation and complex sample preparation, these potentiometric sensors offer rapid, inexpensive, and on-site monitoring, making them highly valuable for routine environmental and food testing.

## 6. Discussion

The comparative evaluation of potentiometric sensors for lead determination is presented in Table 1. The reviewed electrodes were compared in terms of the detection limits, selectivity, and overall performance.

One of the key parameters in sensor performance is the limit of detection (LoD). The solid-contact electrodes, particularly those modified with ionic liquids [9] and BP-C_60_ composites [73], show ultra-low detection limits in the sub-nanomolar range (down to 5.0 × 10^−10^ M), highlighting their suitability for trace-level lead monitoring in complex environmental samples. Similarly, the castor oil-based sensor developed by K. Nisah et al. [64]. achieved an impressive LoD of 10^−10^ M, making it competitive with some stripping voltammetric methods. In contrast, traditional carbon paste electrodes, such as those developed by D. Mishra et al. [67], exhibit higher LoDs (31 μg/L), indicating limited sensitivity for ultra-trace detection but acceptable performance for routine water testing.

The linear response range is another critical factor influencing practical applicability. Most of the studied sensors offer a broad linear range extending over several orders of magnitude. For instance, the electrode developed by C. Wardak exhibited a linear range from 1 × 10^−8^ to 1 × 10^−1^ M, making it suitable for a wide spectrum of contamination levels [9]. Notably, the castor oil-based electrode by Y. Liu et al. displayed a narrower upper detection limit but is designed specifically for saline matrices like seawater, illustrating the trade-off between generality and application-specific optimization [74].

Regarding electrode slope, which indicates Nernstian behavior and sensor responsiveness, most electrodes demonstrated near-Nernstian or slightly sub-Nernstian slopes (~28–30 mV/decade), confirming their effective electrochemical response to Pb^2+^ ions. The slight variation in slope may result from differences in membrane composition, plasticizers, or the use of solid-contact transducers.

The pH operational range varied among sensors, reflecting different tolerance to proton interference and matrix effects. Electrodes like the one by Hussein et al. offered broad pH stability (3–8), making them suitable for food matrices, while others, such as the PANI–PFOA system, function effectively only in narrower pH windows (7–8), suggesting limited versatility but high selectivity under optimal conditions [68].

In terms of sample applicability, the reviewed electrodes cover a range of real-world matrices—from water and wastewater to seawater and food samples. This demonstrates the adaptability of potentiometric methods to diverse analytical challenges. Notably, sensors like those of Li et al. and Wardak have been validated in complex water systems (e.g., river, wastewater [9,73]), underscoring their robustness and environmental relevance. Electrodes tailored for specific matrices (e.g., food samples by Hussein et al. [61]) may require further optimization for broader applicability but provide strong performance in their intended domains. Their recovery rates, ranging mostly from 95% to 105%, indicate the usefulness of the electrodes in the analysis of lead ions.

Finally, innovations in solid-contact materials and membrane modifiers continue to drive progress in potentiometric lead sensing. The incorporation of advanced materials such as BP-C_60_ [73], conducting polymers (e.g., PANI) [74], and ionic liquids significantly improves potential stability and signal reproducibility, and lowers the detection limit. These developments contribute to minimizing calibration frequency and enhance sensor robustness for long-term or field use.

Overall, while classical paste-based electrodes remain attractive for their simplicity and low cost, the latest generations of solid-contact electrodes are clearly outperforming them in terms of analytical sensitivity, stability, and application versatility. Continued efforts to optimize membrane composition, solid-contact interface, and sensor miniaturization will be essential to meet the growing demand for portable, accurate, and calibration-minimal lead sensors in environmental, food, and clinical monitoring.

Pb^2+^ can also be determined using classical, well-defined instrumental methods that have been established as reference techniques in clinical, environmental, and industrial analyses. Table 2 provides a comparative overview of the analytical methods routinely applied by professionals such as ecologists, clinicians, and quality-control specialists for monitoring Pb^2+^ levels in diverse matrices. The table summarizes four representative techniques: graphite furnace atomic absorption spectroscopy (GFAAS), inductively coupled plasma mass spectrometry (ICP-MS), portable anodic stripping voltammetry (ASV), and the NIOSH-approved ultrasonic extraction–ASV protocol for airborne particulates. For each method, typical detection limits (LODs), primary application domains, and references are listed.

A comparison of the values in Table 1 and Table 2 highlights that instrumental methods such as ICP-MS still achieve the lowest detection limits, often at the ng/L level, far surpassing most electrode-based approaches. Similarly, GFAAS achieves sub-µg/L sensitivity in biological samples, making it the clinical reference method for the biomonitoring of lead exposure. However, certain Pb^2+^-selective electrodes (Table 1) already demonstrate LODs in the range of 0.1–1 µg/L, which is comparable to portable ASV systems (~0.2 µg/L, Table 2). This finding is noteworthy, as it shows that well-optimized ISEs can match the performance of some classical electroanalytical methods while offering advantages of portability, simplicity, and low cost.

## 7. Current Limitations and Future Development

### 7.1. Limitations

Despite the notable advantages of potentiometric sensors—such as low cost, simplicity, portability, and real-time detection capabilities—several critical limitations continue to hinder their widespread application, particularly for reliable and ultra-trace lead (Pb^2+^) analysis in complex environments.

While many ion-selective electrodes (ISEs) can achieve detection limits in the micromolar or sub-micromolar range, reaching the parts-per-billion (ppb) levels required for environmental and health standards (e.g., WHO’s 10 ppb lead guideline for drinking water) remains a significant challenge. Sensor performance at low concentrations is often compromised by noise, electrode drift, and interference from background electrolytes [70,81].

Lead-selective ISEs may suffer from cross-sensitivity to other divalent metal ions such as copper (Cu^2+^), cadmium (Cd^2+^), zinc (Zn^2+^), and mercury (Hg^2+^), particularly in environmental or industrial samples with complex ionic compositions. The selectivity (described by selectivity coefficient K, expressed as log K_Pb_^2+^_,j_ where j is an interfering ion) of conventional PVC-based membranes with crown-ether ionophores was shown to be log K_Pb_^2+^_,Na_ = –4.98, log K_Pb_^2+^_,Ca_ = –2.56, log K_Pb_^2+^_,Cu_ = –2.46, and log K_Pb_^2+^_,Cd_ = –2.80, showing that heavy metals such as Cu^2+^ and Cd^2+^ remain problematic interferents [82]. A broader review further confirms that for PVC membranes, selectivity is log K_Pb_^2+^_,Na_ = –3.9, log K_Pb_^2+^_,Ca_ = –6.6, log K_Pb_^2+^_,Mg_ = –6.1, and log K_Pb_^2+^_,Cu_ = –2.1, again emphasizing the need to improve selectivity against copper [2].

While ionophores are designed to favor Pb^2+^ binding, perfect selectivity is rarely achieved, and the presence of interfering ions can lead to inaccurate readings [83].

Many potentiometric sensors—especially those incorporating liquid or plasticized membranes—exhibit limited long-term stability. Plasticizers can leach out over time, and ionophores can degrade due to light, temperature, or chemical interactions. As a result, electrode lifetime and reproducibility are often inferior compared to more robust instrumental techniques like ICP-MS [69]. 

Moreover, ISEs require frequent calibration to maintain accuracy, as their response may drift over time due to changes in membrane composition, electrode fouling, or environmental conditions. This drift complicates long-term or unattended monitoring applications and can limit the practicality of the sensors in remote or resource-limited settings [2,84]. For example, disposable, plasticizer-free solid-contact ISEs based on MWCNTs exhibited no obvious potential drift when exposed to O_2_ and CO_2_, highlighting their excellent stability under ambient conditions [85].

Although modern ISEs have improved response times, hysteresis effects and slow stabilization in low-ionic-strength or high-resistance samples can still occur. This can delay measurement processes and affect the consistency of results during rapid or high-throughput analyses [86].

While there is growing interest in miniaturized and wearable potentiometric devices, integrating lead-selective membranes into stable, reproducible microfabricated platforms remains difficult. Issues such as miniaturized reference electrode stability, membrane adhesion, and interface noise become more significant as device dimensions shrink [86]. The comparative data underscore that while nanocomposite SC-ISE designs can minimize drift, there remains a significant need to optimize ionophore chemistry for enhanced discrimination against chemically similar divalent metals.

### 7.2. Opportunities for Future Development

The detection of lead (Pb^2+^) in environmental and biological samples continues to be a critical challenge that calls for innovative sensor technologies. While potentiometric sensors have demonstrated significant potential due to their simplicity and portability, future developments focused specifically on lead sensors can greatly enhance sensitivity, selectivity, and practical deployment [41].

Designing highly selective ionophores specifically tuned for Pb^2+^ remains a key opportunity. Emerging materials like molecularly imprinted polymers (MIPs) and novel organic ligands can provide superior affinity and discrimination against competing metal ions such as Cd^2+^ and Zn^2+^, thereby improving lead-specific response and reducing false positives [12,87].

Incorporating nanomaterials such as graphene, carbon nanotubes, and metal nanoparticles into lead sensor designs can substantially improve detection limits and sensor responsiveness. Nanostructured surfaces enhance electron transfer and increase active sites for lead binding, making sensors more sensitive even at ultra-trace lead concentrations [88,89,90].

Advancing stable, miniaturized solid-contact electrodes tailored for lead detection can facilitate portable and wearable sensor devices. Innovations in solid-contact materials and reference electrodes that maintain stability specifically in lead-rich matrices will improve long-term reliability and reduce calibration needs.

Combining potentiometric lead sensors with complementary techniques—such as voltammetry, fluorescence, or colorimetric detection—can create hybrid platforms that offer enhanced accuracy and robustness. Multiplexed arrays capable of the simultaneous detection of lead and other heavy metals would be valuable for comprehensive environmental monitoring [91,92].

The development of wearable or deployable lead sensors capable of real-time in situ monitoring in biological fluids (e.g., sweat, blood) or environmental waters could revolutionize exposure assessment and public health. Recent studies demonstrate that potentiometric and related ion-selective sensing platforms can be adapted for the real-time monitoring of Pb^2+^ in environmental systems. Micro-scale ISEs have enabled the continuous in situ profiling of lead release from plumbing materials, with stable operation over more than 16 h, providing valuable insights into dynamic leaching processes [93]. In wastewater applications, screen-printed solid-state ion-selective membranes equipped with algorithm-based pH self-correction have achieved the long-term continuous monitoring of Pb^2+^ in the range of tens to hundreds of ppb [94]. Furthermore, chemiresistive ion-selective sensors with Pb^2+^-selective membranes have recently been reported for *online* monitoring, reaching detection limits below 2 µg L^−1^ and covering a concentration range up to 3 mg L^−1^ [95]. These examples highlight a clear trend towards the development of real-time and field-deployable lead sensors, which could complement advanced laboratory-based techniques and expand the applicability of potentiometric sensing in routine environmental monitoring.

Integration with wireless data transmission and smartphone interfaces would enable rapid, on-site decision making [86]. Finally, reducing the need for frequent calibrations is crucial for practical lead sensor applications, especially in long-term and remote monitoring. Future developments aimed at improving the inherent potential stability of lead-selective electrodes—through more stable membrane compositions, solid-contact materials, and robust reference electrodes—can significantly limit signal drift. Coupling these hardware improvements with smart calibration algorithms and real-time drift compensation will enable more reliable, maintenance-free operation and expand the usability of lead sensors in diverse field conditions.

## 8. Conclusions

Lead remains a widespread and toxic environmental contaminant with significant health implications, especially due to its bioaccumulative nature and harmful effects on the nervous, cardiovascular, and renal systems. The need for rapid, sensitive, and field-deployable methods for lead ion (Pb^2+^) detection continues to grow, particularly for monitoring drinking water, food products, industrial effluents, and biological samples.

Potentiometric methods, while often overshadowed by techniques like AAS or ICP-MS, offer distinct advantages, such as simplicity, low cost, portability, and rapid response, without the need for complex instrumentation or extensive sample preparation. These attributes make them highly suitable for on-site and real-time lead monitoring, especially in resource-limited settings.

A range of electrode designs has been reviewed, from conventional carbon paste systems to advanced solid-contact configurations modified with ionic liquids, conducting polymers, and nanomaterials. The comparison of selected sensors revealed remarkable progress in lowering detection limits down to the sub-nanomolar range, broadening linear ranges, enhancing selectivity, and enabling use in diverse sample types including wastewater, seawater, and food matrices. 

Despite these advancements, several limitations still slow down the widespread adoption of potentiometric lead sensors. Challenges such as long-term potential drift, miniaturized reference electrode stability, membrane adhesion, and susceptibility to sample matrix effects remain areas of concern. Furthermore, narrow optimal pH windows and potential interferences from coexisting ions can limit the versatility of certain designs.

Looking ahead, the future development of lead-selective sensors will likely focus on creating highly selective ionophores, improving membrane materials, and integrating nanostructures to enhance signal transduction. The move toward solid-contact, calibration-free, and wireless sensor systems—possibly with smartphone integration—opens the door to practical solutions for real-time monitoring. Importantly, improving the potential stability of these sensors will be key to reducing the frequency of calibration and supporting long-term deployment in field conditions.

In summary, potentiometric sensors—especially those employing modern materials and design strategies—offer a compelling approach to selective and sensitive lead detection. With continued interdisciplinary research and engineering innovations, these devices can evolve into powerful tools for environmental safety and public health.

## Figures and Tables

**Table 1:** Overview of potentiometric sensor technologies for lead monitoring in real-life samples.

Electrode Design	Limit of Detection [M]	Linear Range [M]	Slope [mV/dec]	Working pH Range	Sample Type for Lead Detection	Recovery Rates [%]	Reference
castor oil-based electrode with internal solution	10^−10^	1.0 × 10^−10^–1.0 × 10^−5^	27.25	7–8	wastewater	N/R	[64]
composite carbon paste electrode	1.5 × 10^−7^	9.7 × 10^−10^–7.2 × 10^−7^	N/R	1–5	water	N/R	[67]
graphite paste electrode with TCP as plasticizer	3.33 × 10^−5^	1.0 × 10^−5^–1.0 × 10^−2^	29.50 ± 0.40	3–8	food samples	97.6–101.2	[68]
graphite paste electrode with o-NPOE as plasticizer	3.33 × 10^−6^	1.0 × 10^−6^–1.0 × 10^−2^	29.90 ± 0.56	3–9	food samples	97.3–101.8	
solid-contact electrode modified with ionic liquids	4.3 × 10^−9^	1.0 × 10^−8^–1.0 × 10^−1^	29.8	3.5–7.3	tap, river and wastewater	92.7–104.8	[9]
solid-contact electrode modified with NC	6.0 × 10^−9^	1.0 × 10^−8^–1.0 × 10^−2^	31.5	3.1–7.6	wastewater	N/R	[72]
BP-C_60_-based solid-contact electrode	5.0 × 10^−10^	1.0 × 10^−9^–1.0 × 10^−3^	28.8 ± 1.2	N/R	tap water	N/R	[73]
PANI–PFOA solid-contact electrode with PAMTB additive	1.9 × 10^−7^	1.0 × 10^–6^–1.0 × 10^−3^	28.5 ± 0.8	N/R	seawater	89.5–110.9	[74]

N/R—not reported. LoD and linear range units were converted to molarity [M] for consistency.

**Table 2:** Classical analytical methods widely applied for Pb^2+^ monitoring in clinical, environmental, and industrial contexts. The table summarizes typical detection limits, and common application domains.

Method	Average Detection Limit	Use Case/Professional Application	Reference
Graphite Furnace AAS (GFAAS)	1.6 µg/L in blood	Clinical diagnostics (biomonitoring of Pb exposure)	[77]
ICP-MS (iCAP TQ)	1 ng/L	High-sensitivity environmental and quality control analysis	[78]
Portable ASV (scTRACE Gold)	0.2 µg/L	Field monitoring of drinking water	[79]
Ultrasonic Extraction + ASV (NIOSH 7701)	0.05 µg per air sample	Occupational/environmental health monitoring (air filters)	[80]

## Data Availability

No new data were created or analyzed in this study. Data sharing is not applicable to this article.

## Abbreviations

The following abbreviations are used in this manuscript:

AAS	Atomic Absorption Spectrometry
ASV	Anodic Stripping Voltammetry
BBPA	Bis(1-butylpentyl) adipate
BMImCl	1-butyl-3-methylimidazolium chloride
BP C_60_	Bimodal Pore C_60_
CNF	Carbon Nanofibers
CPE	Carbon Paste Electrode
DMImCl	1-decyl-3-methylimidazolium chloride
DOS	Dioctyl Sebacate
EMF	Electromotive Force
EMImCl	1-ethyl-3-methylimidazolium chloride
ETH 500	tetradodecylammonium tetrakis-(4-chlorophenyl)borate
GC	Glassy Carbon
GFAAS	Graphite Furnace Atomic Absorption Spectrometry
HMImCl	1-hexyl-3-methylimidazolium chloride
HPIMN	Novel Schiff Base Ligand Ionophore
HMImPF_6_	1-hexyl-3-methylimidazolium hexafluorophosphate
ICP MS	Inductively Coupled Plasma Mass Spectrometry
ISE	Ion-Selective Electrode
KTpClPB	Potassium-tetrakis-(4 chlorophenyl)borate
LoD	Limit of Detection
MIP	Molecularly Imprinted Polymer
NaTFPB	Sodium trakis[3,5-bis(trifluoro-methyl)phenyl]borate
NC	Nanocomposite
NIOSH	National Institute for Occupational Safety and Health
NMImCl	1-benzy-3-methylimidazolium chloride
o NPOE	2-nitrophenyl Octyl Ether
PAMTB	Propyl 2-(acrylamidomethyl)-3,4,5-trihydroxy benzoate
PANI	Polyaniline
PEDOT	Poly(3,4-ethylenedioxythiophene)
PFOA	Perfluorooctanoic Acid
PU	Polyurethane
PVC	Polyvinyl Chloride
PSS	Poly(styrene sulfonate)
TDI	Toluene Diisocyanate
TBP	Tributyl phosphate
THF	Tetrahydrofuran
TMTDS	Tetramethyl Thiuram Disulfide
TCP	Tricresylphosphate
WHO	World Health Organization
